# 

**Published:** 1898-08

**Authors:** 


### CONTENTS.

COMMUNICATIONS.

The Essential Oils and Some Other Agents—Their Antiseptic Value; also, Their Irritating or Non Irritating Properties.—Abstracts. 351
Dental Caries...... 368
Some Facial Deformities and Prevention.—Abstract ...... 386

COMMENCEMENTS.

Southern Medical College—Dental Department ...... 390
New York College of Dentistry ...... 390
Birmingham Dental College ...... 391
Northwestern College of Dental Surgery ...... 391
Tacoma College of Dental Surgery ...... 392
Royal College of Dental Surgeons of Ontario ...... 392
Missouri Dental College...... 393
National University—Dental Department...... 394
University of Colorado—Dental Department ...... 394
Baltimore Medical College—Dental Department...... 394
New York Dental School...... 395
Central College of Dentistry...... 395
Washington Dental College ...... 396
Howard University—Dental Department...... 396
University of Denver—Dental Department...... 396

EDITORIAL

Professional Recruits ...... 397
The Bi-State Dental Meeting ...... 399
To the Omaha Meetings...... 399
Annual Meeting of the National Association of Dental Faculties..... 400
National Dental Association...... 400

